# Threading the Needle in Bladder Cancer: A Narrative Review of the Emerging Role of Integrative Oncology in Treatment-Related Adverse Events in the Era of Immunotherapy and Antibody-Drug Conjugates

**DOI:** 10.7759/cureus.111294

**Published:** 2026-06-22

**Authors:** Arman M Niknafs, Leland F Damron, Nikita V Baclig, Marilena Iliopoulos, Alexandra Drakaki

**Affiliations:** 1 Department of Medicine, University of California Los Angeles David Geffen School of Medicine, Los Angeles, USA; 2 Department of Medicine, Division of Hematology/Oncology, University of California Los Angeles David Geffen School of Medicine, Los Angeles, USA

**Keywords:** acupuncture, antibody drug conjugates, bladder cancer, chemotherapy neuropathy, immune checkpoint inhibitors, integrative oncology, quality of life, supportive oncology, survivorship care, treatment toxicity

## Abstract

Bladder cancer is a common malignancy with substantial morbidity, and recent advances in immune checkpoint inhibitors, antibody-drug conjugates, and targeted therapies have improved outcomes across disease stages. Despite these gains, emerging therapies are associated with substantial treatment-related adverse events (TRAEs), including neuropathy, rash, gastrointestinal, ocular, and immune-mediated toxicities, often leading to dose reductions or therapy discontinuation. In this review, we aim to examine the landscape of integrative oncology, its areas of efficacy and uncertainty, and how these therapies may mitigate TRAEs in bladder cancer survivors. Integrative oncology provides patient-centered, evidence-informed approaches to alleviate TRAEs and enhance quality of life. Evidence from randomized trials and systematic reviews suggests that a range of integrative oncology interventions, including acupuncture, cryotherapy, and mind-body therapies, may help temper treatment-related symptoms and improve quality of life among cancer survivors. Nutritional and lifestyle interventions may offer additional benefits, although evidence regarding their safety and optimal implementation remains limited. While most supporting evidence derives from non-genitourinary malignancies, important differences in treatment regimens, patient characteristics, and toxicity profiles limit direct extrapolation to bladder cancer populations, despite overlap in key symptom domains such as fatigue, pain, and peripheral neuropathy. Prospective, disease-specific research is needed to define best practices and integrate multidisciplinary supportive care with conventional therapies. Thoughtful incorporation of integrative oncology may help “thread the needle” between treatment intensity and patient-centered care, with the potential to enhance adherence, survivorship, and the overall patient experience.

## Introduction and background

Bladder cancer is the fourth most common malignancy and the 10th leading cause of death in the United States, with an estimated 85,000 new cases in 2026 [1]. Over the past decade, substantial advances in drug development have transformed the management of bladder cancer and have contributed to significant improvements in mortality rates [2]. Treatment has entered a dynamic era marked by the rapid expansion of immunotherapies, targeted agents, and innovative intravesical and drug delivery approaches aimed at improving outcomes across disease stages [3,4]. However, with emerging therapies, there is a risk of novel and clinically significant treatment-related adverse events (TRAEs). This review aims to "thread the needle" by examining the evolving spectrum of TRAEs in bladder cancer and the emerging role of integrative oncology, including acupuncture, as a patient-centered, evidence-based strategy for supportive care [5,6]. 

Traditional platinum-based chemotherapy remains foundational for many patients with bladder cancer, with TRAEs that are well recognized and routinely anticipated in clinical practice. Modern treatment paradigms have moved towards the incorporation of immune checkpoint inhibitors (ICIs) such as pembrolizumab, nivolumab, avelumab, atezolizumab, and durvalumab, in perioperative, adjuvant, first-line metastatic, or maintenance settings [3,4]. Although the toxicity profile of ICIs is widely described and largely driven by inflammation-mediated processes such as pneumonitis, colitis, hepatitis, and endocrinopathies, novel toxicity syndromes continue to emerge [7]. For instance, Triple M syndrome, characterized by the co-occurrence of myocarditis, myositis, and myasthenia gravis, has only recently been described [8].

Beyond ICIs, novel antibody-drug conjugates (ADCs) and small-molecule therapies with novel molecular targets are reshaping the treatment landscape of both early-stage and metastatic bladder cancer, but introduce new and clinically significant TRAEs. Enfortumab vedotin was the first approved ADC that targets nectin-4 and is already in wide use in the perioperative and metastatic setting [9,10]. Additional ADCs, such as disitamab vedotin targeting human epidermal growth factor receptor 2 (HER2) and datopotamab deruxtecan targeting trophoblast cell surface antigen-2 (TROP-2), are well into development, while sacituzumab govitecan, another TROP2 ADC, is available even if its accelerated approval has been voluntarily withdrawn [11]. This class of drugs is associated with distinct toxicity profiles compared to chemotherapy and ICIs, which is due to either the payload utilized or the target itself. The most commonly reported adverse events (AEs) include, but are not limited to, rash, sensory and/or motor neuropathy, diarrhea, cytopenias, as well as metabolic and ocular toxicities [12]. Furthermore, targeted therapies that inhibit fibroblast growth factor receptor (FGFR), such as the on-market erdafitinib or the investigational agent vepugratinib, uniquely drive hyperphosphatemia, oral and mucosal toxicity, as well as gastrointestinal side effects [13,14]. As novel therapies are increasingly incorporated into bladder cancer treatment paradigms, effective management of TRAEs has become an important component of both balancing treatment tolerability and optimizing patient outcomes.

Methodology

To inform this review, a structured search of PubMed and ClinicalTrials.gov was performed, supplemented by manual review of reference lists, relevant clinical practice guidelines, and expert input from genitourinary oncologists at the University of California, Los Angeles (UCLA), California, United States. The search proceeded through several phases: identification of society-level integrative oncology guidelines from National Comprehensive Cancer Network (NCCN), Society for Integrative Oncology (SIO), and American Society of Clinical Oncology (ASCO); review of the definitions, mechanisms, and clinical applications of individual integrative modalities; assessment of evidence quality with emphasis on randomized controlled trials, systematic reviews, and meta-analyses; characterization of adverse event profiles associated with contemporary ICIs, ADCs, and targeted therapies as reported in recent bladder cancer trials; and evaluation of integrative oncology implementation for TRAE management across cancer types, with particular attention to bladder cancer-specific data. Search terms included combinations of "integrative oncology," "acupuncture," "complementary therapy," "treatment-related adverse events," "bladder cancer," "urothelial carcinoma," "immune checkpoint inhibitors," "antibody-drug conjugates," and related terms. Articles published through 2026 were considered. No formal systematic review methodology was applied, consistent with the narrative design of this review.

## Review

TRAEs in contemporary bladder cancer trials

Inevitably, irrespective of the drug being used, dose reductions, delays, and complete discontinuation due to intolerance are not uncommon. For example, in the landmark trial EV-302, which led to the approval of enfortumab vedotin with pembrolizumab in the first-line setting, AEs led to therapy discontinuation in 35% of patients in the combination group, compared to just 19% in the platinum-based chemotherapy group [9,10].

This finding was also noted across numerous bladder cancer trials. TROPiCS-04 analyzed the anti-tumor activity of sacituzumab govitecan (SG) versus chemotherapy [15]. While there was a higher objective response rate (ORR), there was no overall survival benefit, possibly because of early discontinuation of SG due to toxicity. CheckMate 901 demonstrated significantly better outcomes in patients treated with Nivolumab and Gemcitabine-Cisplatin compared to platinum-based chemotherapy alone [16]. However, safety analysis revealed that grade 3 or higher TRAEs and discontinuation occurred in 77% and 21% of patients treated with Nivolumab and chemotherapy, respectively, compared to 68% and 17% in the chemotherapy-only group. Further details on major bladder cancer trials and associated TRAEs are provided in Table 1.

**Table 1:** Rates of Dose Reduction, Modification, and Discontinuation due to Treatment-Related Adverse Events in Landmark Bladder Cancer Trials *Treatment of physician’s choice: paclitaxel 175 mg/m2, docetaxel 75 mg/m2, or vinflunine 320 mg/m2 [15]

Trial name	Phase	Agent(s)	N	Grade 3 or Higher TRAEs (%)	Dose Reduction or Modification due to TRAEs (%)	Termination due to TRAEs (%)
EV-302 [10]	III	Enfortumab Vedotin + Pembrolizumab	442	56	41	35
		Platinum-based chemotherapy	444	70	38	19
TROPiCS-04 [15]	III	Sacituzumab Govitecan	349	67	37	11
		TPC*	337	35	26	12
CheckMate 901 [16]	III	Nivolumab + Gemcitabine- Cisplatin	304	77	NR	21
		Gemcitabine- Cisplatin	288	68	NR	17
KEYNOTE-361 [17]	III	Pembrolizumab + Chemotherapy	351	87	NR	31
		Pembrolizumab	307	63	NR	16
		Chemotherapy	352	82	NR	18
IMvigor130 [18]	III	Atezolizumab	354	46	36	9
		Placebo + Chemotherapy	389	50	78	8
DANUBE [19]	III	Durvalumab	346	14	28	12
		Durvalumab + Tremelimumab	342	27	36	24
		SOC Chemotherapy	344	60	69	17
BLC2001 [13]	II	Erdafitinib	99	66	56	13

More than 90% of patients across the majority of multicenter bladder cancer trials had at least a grade 1 TRAE. Commonly reported toxicities include peripheral sensory neuropathy and alopecia [10], pruritus and rash [17], fatigue [10,16,17], as well as immune-related adverse events (IrAEs) when ICIs are used [7]. While the discontinuation of therapy is, at times, inevitable for life-threatening adverse effects, some symptoms may be temporized if addressed correctly and proactively.

The high rate of treatment interruptions and premature cessation due to TRAEs in bladder cancer trials presents an opportunity to explore new avenues for symptom management and, subsequently, increase time under treatment to improve patient outcomes. One of these avenues is the incorporation of integrative oncology into the treatment plan and, thereby, empowering patients to take an active role in their care.

Integrative oncology: modalities and rationale

Integrative oncology is a discipline that combines mind and body practices, natural products, and lifestyle interventions with conventional cancer treatments to enhance patient health, quality of life, and clinical outcomes across the continuum of care. It is contingent on being patient-centered, actively engaging cancer survivors as partners in their care, and promoting multimodal care implementation throughout the cancer journey [5,6,20].

Acupuncture is among the most well-studied and widely implemented integrative oncology modalities, with applications in managing pain, nausea, fatigue, hot flashes, and anxiety related to cancer and its treatment [21,22]. Acupressure involves the application of manual pressure rather than needles; as a non-pharmacological, patient-directed technique, it may offer broader accessibility for enabling self-care alongside systemic therapies [23]. 

Cryotherapy leverages local vasoconstriction to reduce drug exposure at vulnerable sites [24]. Applications include utilizing temperature-controlled gloves and socks to mitigate symptoms of chemotherapy-induced peripheral neuropathy (CIPN), oral cryotherapy (e.g., chewing ice cubes) for chemotherapy-induced mucositis prevention, and cooling eye masks aimed at reducing corneal toxicity, a common ADC-associated TRAE [25-27]. With three automated- and FDA-approved devices available, scalp cooling has been shown to attenuate symptoms of chemotherapy-induced alopecia [28,29].

Mind-body practices, ranging from meditation to yoga and traditional Chinese practices of Tai Chi/Qigong (TCQ), are utilized as complementary modalities targeting cancer-related pain, emotional distress, fatigue, sleep disturbance, and overall well-being. These practices share a foundation in cultivating present-moment awareness and integrating controlled movement or breathwork and collectively represent a patient-centered foundation for symptom management from diagnosis to survivorship [30-32]. 

Nutritional considerations are increasingly recognized as a component of comprehensive cancer care. Dietary optimization and evidence-informed supplementation, such as antioxidants, herbs, fiber, and vitamins, may play a supportive role in multidisciplinary oncology practice. However, patients should be cautioned against excessive or unregulated supplement use given growing concerns regarding the lack of independent safety and potency testing, potential drug-supplement interactions, and unanticipated adverse effects [33].

Evidence for integrative oncology in cancer-related symptom management

In recent years, integrative oncology, which encompasses a number of mind-body, manual, and physical modalities, has moved from the periphery into formal, guideline-level recommendations meant to be implemented alongside conventional cancer care. This evolution is demonstrated by the emergence of codified recommendations from the NCCN as well as a collaboration between the SIO and the ASCO, where integrative modalities are presented as options to manage physical and psychological sequelae of cancer treatment [34-36].

Evidence from Non-Bladder Cancer Populations 

The strongest evidence base for integrative oncology comes from patients with solid tumors such as breast and lung cancer. Over the last decade, numerous prospective RCTs designed to test the efficacy of the various aforementioned interventions and systematic reviews assessing the impact of their outcomes have emerged in these populations [37-39]. These trials have studied outcomes ranging from CIPN and fatigue to pain, insomnia, anxiety, depression, and overall quality of life.

Acupuncture is among the integrative oncology modalities with the strongest supporting evidence, particularly for the management of CIPN-related symptoms. Interpretation of the literature, however, is complicated by heterogeneity in study design, patient populations, treatment schedules, and the use of adjunctive techniques such as electrical stimulation and moxibustion. Nevertheless, a 2024 meta-analysis including 33 RCTs and over 2000 patients, primarily involving non-genitourinary malignancies, demonstrated that acupuncture-related practices significantly improved CIPN pain symptoms and quality of life [37]. Standard acupuncture was shown to be most effective in reducing CIPN pain, while protocols combining standard treatment with electric stimulation or moxibustion revealed the greatest quality of life improvements. A 2020 RCT by Bao et al. conducted at Memorial Sloan Kettering Cancer Center, New York, United States, compared eight weeks of standard acupuncture to sham or usual care in 75 patients and found that real acupuncture produced greater reductions in neuropathic symptom severity than sham or usual care, thus supporting a non-placebo effect [39].

Cryotherapy represents one of the more evidence-supported integrative interventions, particularly in reducing the severity of chemotherapy-induced mucositis, where its use in randomized trials using 5-fluorouracil (5-FU) and high-dose melphalan-based regimens has shown consistent benefit [40,41]. In contrast, data supporting cryotherapy for the prevention of CIPN remain heterogeneous and are largely confined to non-genitourinary malignancies [25]. As a result, the applicability to bladder cancer populations is limited (though 5-FU may be used as a radiosensitizer in certain protocols) given the evolution of contemporary treatment paradigms.

Beyond manual therapies, mind-body and movement-based practices such as TCQ and yoga have also generated evidence for improving survivorship outcomes. A meta-analysis examining 15 RCTs including 1283 patients (drawing from breast, prostate, mixed-solid cancer, and lymphoma cohorts) found significant improvements in fatigue, sleep difficulty, depression, and overall well-being [32]. Similarly, yoga has been highlighted in recent trials, namely the nationwide multicenter YOCAS^©®^ (Yoga for Cancer Survivors) RCT, which showed potential in reducing cancer-related fatigue and improving sleep quality following a standardized, four-week yoga intervention [42]. Randomized trials of mindfulness-based stress reduction (MBSR) in cancer survivor cohorts in the United States, predominantly outside bladder cancer populations, have demonstrated meaningful reductions in emotional distress and sleep disturbances while improving quality of life [43,44].

In addition to the mind-body and movement practices discussed above, structured exercise programs incorporating aerobic and resistance training have emerged as a distinct and robustly supported integrative oncology modality. ASCO and NCCN guidelines recommend aerobic and combined aerobic-resistance exercise during and after treatment to reduce the effects of treatment-associated anxiety, depression, fatigue, and functional decline [45-47].

Bridging to Bladder Cancer 

The evidence supporting these interventions has been primarily generated in breast, thoracic, and mixed-solid tumor populations, limiting direct extrapolation to patients with bladder cancer. Despite this expanding knowledge base, notable gaps persist within genitourinary cancers, and in particular, bladder cancer. In contrast to breast and thoracic oncology, integrative approaches targeting genitourinary cancer remain markedly understudied. To date, no prospective RCTs have specifically evaluated integrative oncology modalities alongside contemporary bladder cancer treatments, with the notable exception of prehabilitation before radical cystectomy. Here, a 2025 scoping review identified 24 RCTs (n=2,471 participants) in which 71% of tested interventions (e.g., exercise, psychological support, nutrition) demonstrated significant prehabilitation-associated improvements in domains such as strength, functional performance, and body composition; furthermore, a complementary meta-analysis confirmed that preoperative exercise improved post-cystectomy quality of life [48,49]. These findings are being further evaluated in ongoing multicenter RCTs, including the ENHANCE trial (NCT05480735), a multimodal prehabilitation study integrating exercise, nutrition, and psychological support across eight centers in the Netherlands, and the Get Moving Trial (NCT06040762), a phase I/II RCT evaluating a pragmatic, app-based home exercise program during neoadjuvant chemotherapy and through 90 days post cystectomy [50,51].

Importantly, the symptom domains addressed by prehabilitation and integrative oncology closely overlap with the predominant TRAEs of ADCs and ICIs, which are increasingly central to bladder cancer care. Peripheral neuropathy is the most clinically impactful ADC-related toxicity in this space, as shown in EV-302 [10]. This trial demonstrated that peripheral sensory neuropathy occurred in 50% of patients who received enfortumab vedotin plus pembrolizumab, was the leading cause of dose reduction (10%) and discontinuation (5%), and generated persistent, residual neuropathy at last follow-up for 87% of affected participants. This ADC-driven neuropathy burden directly mirrors the symptom domains where acupuncture and physical exercise demonstrate the most consistent benefits. A recent meta-analysis investigating non-pharmacologic treatments to mitigate CIPN symptoms, which included 27 RCTs and a total of 2,136 patients, found that physical exercise provided statistically significant alleviation of neurotoxicity symptoms compared to medication alone, while acupuncture reduced neuropathic pain and CIPN incidence [52]. Cancer-related fatigue, another prevalent TRAE reported in 29-51% of patients across EV-302 [10], CheckMate 901 [16], and KEYNOTE-361 [17], similarly aligns with the integrative oncology domains carrying the strongest guideline-level endorsement, as NCCN guidelines list physical activity, yoga, and massage therapy as Category 1 recommendations during active treatment [46].

Stemming from their role in mitigating individual symptoms, integrative modalities, particularly physical activity, may also influence ICI-specific outcomes more broadly. This is of particular relevance as ICIs become embedded across perioperative, adjuvant, maintenance, and first-line metastatic bladder cancer settings. A prospective cohort study of 251 patients receiving anti-PD-(L)1 therapy (UNICIT) found a dose-dependent relationship between physical activity and immune-related toxicity, where severe TRAE rates within one year declined from 29% in the low activity group to 14% and 10% in the moderate and high activity groups, respectively. High physical activity levels were associated with approximately an 80% reduction in the odds of developing severe TRAEs and nearly a 50% improvement in overall survival compared to the least active patients [53]. These results were supported in a larger retrospective cohort study of 930 ICI-treated patients, where higher physical activity levels were associated with an 11% reduction in the hazard of death per increasing activity quartile [54]. However, given the retrospective design and reliance on self-reported physical activity, these findings are subject to residual confounding and should be interpreted as associative rather than causal. Collectively, these data suggest that integrative modalities, including exercise and acupuncture, may be associated with outcomes relevant to treatment tolerability in contemporary bladder cancer care, which support further disease-specific investigation.

While exercise represents one avenue through which integrative oncology may show promise in managing emerging TRAEs, early efforts have also explored pharmacologically oriented complementary approaches in this space. A recent systematic review and meta-analysis assessing the impact of traditional Chinese medicine (TCM) combined with ICIs across several cancer types reported possible improvements in response rates and in some TRAEs; however, the included studies were generally small, single-center trials with unclear risk of bias, inconsistent blinding, and poor herbal formulation standardization [55]. Notably, none of these studies included patients with bladder cancer, which is consistent with the broader evidence gap in genitourinary cancer noted above, and with all studies taking place in China, the findings have constrained applicability to United States settings where population demographics, regulatory oversight, patient perceptions, and the supportive care infrastructure may differ substantially.

These limitations reflect broader methodological challenges in the integrative oncology literature that meaningfully influence the interpretation of reported effects. Across modalities, many studies remain underpowered and are limited by inconsistent blinding, variable standardization of interventions, and heterogeneous outcome measures, all of which increase the risk of bias and reduce comparability across trials. Consequently, reported effects should be interpreted cautiously, particularly when extrapolating across cancer types and clinical settings. The systematic review by Yeh et al., which evaluates acupuncture for CIPN, similarly reports concerns regarding selection bias, unclear blinding and randomization, and limited generalizability beyond East Asian practice contexts [37]. Beyond problematic design elements, safety considerations for TCMs in particular require attention as well, namely whether supplements pose clinically significant herb-drug interactions, deleterious immunomodulatory effects for ICIs, and issues related to regulations around product quality and consistency.

Beyond these methodological considerations, practical implementation barriers may further limit the real-world adoption of integrative oncology. Formal programs remain concentrated at larger, not-for-profit, metropolitan teaching institutions, and many cancer centers lack integrative medicine providers with the oncology-specific competency needed to safely treat medically complex patients. Financial barriers also compound these access challenges. Most integrative modalities are not routinely covered by insurance, and out-of-pocket costs may be prohibitive for patients already experiencing cancer-related financial toxicity. Adherence may be further limited by the practical demands of prolonged bladder cancer treatment, as time constraints, scheduling logistics, and geographic distance from specialized providers collectively contribute to attrition over time, while provider-level knowledge gaps may limit referral and uptake [56].

## Conclusions

The therapeutic landscape of bladder cancer has evolved substantially with the implementation of ICIs, ADCs, and targeted therapies, which have improved outcomes across disease stages. However, these gains are accompanied by a substantial burden of TRAEs that compromise patient quality of life, treatment adherence, and their ability to remain on life-prolonging therapies. As contemporary trials consistently demonstrate, toxicity-related dose reductions and discontinuations remain a defining challenge even as efficacy improves with advanced therapies. Integrative oncology, with a particular focus on acupuncture, mind-body practices, and structured exercise, offers a promising and evidence-informed framework to meet this need. Thoughtfully embedding these modalities within conventional care has the potential to improve patient experience, treatment tolerability, and potentially extend time on therapy. 

Moving forward, prospective bladder cancer-specific trials that weave multidisciplinary supportive care components into standard treatment pathways will be essential to define best practices and clarify which integrative approaches are most effective alongside evolving systemic therapies, such as structured exercise programs, acupuncture delivered within supportive care services, and other symptom-directed approaches integrated into existing infusion or perioperative workflows. Trial designs should prioritize real-world feasibility, using pragmatic or platform-based approaches that leverage existing clinical services and infrastructure. Key endpoints should include patient-reported outcomes (fatigue, neuropathy, quality of life), functional measures, and clinically relevant treatment delivery outcomes such as dose reductions, delays, and discontinuation, alongside systematic assessment of TRAEs.
